# Field Efficacy of *Steinernema* sp. (Rhabditida: Steinernematidae) on the Colorado Potato Beetle Overwintering Generation

**DOI:** 10.3390/plants10071464

**Published:** 2021-07-16

**Authors:** Maja Čačija, Renata Bažok, Majda Kolenc, Tena Bujas, Zrinka Drmić, Martina Kadoić Balaško

**Affiliations:** 1 Department of Agricultural Zoology, Faculty of Agriculture, University of Zagreb, Svetošimunska 25, 10000 Zagreb, Croatia; rbazok@agr.hr (R.B.); majdakolenc9@gmail.com (M.K.); tena.bujas27@gmail.com (T.B.); mbalasko@agr.hr (M.K.B.); 2 Croatian Agency for Agriculture and Food, Plant Protection Center, Vinkovačka cesta 63c, 31000 Osijek, Croatia; zrinka.drmic@hapih.hr

**Keywords:** biological control, entomopathogenic nematodes, *Leptinotarsa decemlineata*, *Steinernema carpocapsae*, *Steinernema feltiae*

## Abstract

Colorado potato beetle (CPB) is an economic pest of potato that has developed resistance to all classes of chemical insecticides, thus requiring alternative control measures. As a potential solution, entomopathogenic nematodes (EPNs) have proven effective in suppressing this pest, but their efficacy against overwintering generations of CPB in Croatia has not been sufficiently researched. The aim of this two-year (2018–2019) field study was to determine the efficacy of *Steinernema feltiae* and *Steinernema carpocapsae* applied to overwintering CPB adults. EPNs were applied at three doses (7.5 mil./10 m^2^, 5.0 mil./10 m^2^ (the recommended dose) and 2.5 mil./10 m^2^) by watering the soil where the adults were overwintering. The first-year results were satisfactory for both EPNs: the efficacy of *S. feltiae* ranged from 79.03% to 100.00%, while the efficacy of *S. carpocapsae* ranged from 77.32% to 96.22%. In the second year, the highest efficacy (69.57%) was obtained using the recommended dose of *S. feltiae*. Although the results are not consistent across the two years of our study and suggest further research, they indicate that EPNs have great potential in controlling overwintering CPB generations to reduce first generation abundance and damage, and also to prevent the spread of new generations to surrounding potato growing areas.

## 1. Introduction

The Colorado potato beetle (*Leptinotarsa decemlineata* (Say); CPB) is the most important defoliating pest of potatoes [1]. It is widely distributed in North America, Europe, and Asia, covering an area of about 16 million km^2^, and continues to spread [2]. In the 20th century, this pest has become a major problem throughout Europe, Asia, and China [3]. For successful potato production, CPB was controlled with insecticides. Initially, insecticides had great efficacy against CPB. The first recorded case of resistance was in the 1950s, against DDT [2]. Over time, CPB developed resistance to the newly registered insecticides which were initially effective [1]. Insecticide resistance is a major problem in crops, landscapes, and indoor environments [4,5]. To date, more than 300 cases of CPB resistance to 56 insecticidal active ingredients have been detected [6].

Resistance to chlorinated hydrocarbons (DDT, aldrin, and dieldrin) was first reported in EU countries [7]. Resistance of CPB to the active ingredients DDT and lindane was detected in Croatia in 1967, and resistance to organophosphorus insecticides, carbamates and pyrethroids was also reported in the late 1980s [7]. The main reason for the rapid spread of CPB resistance was improper use, i.e., exceeding prescribed doses, use of insecticides with the same mode of action, and frequent application [7]. Similar factors affecting the development of CPB resistance were previously reported by Alyokin et al. [2]. The problem with developing new insecticides and creating new products that are effective and prevent the emergence of resistance is that it is time-consuming and costly to do so [2]. In order to prevent the emergence of resistance (anti-resistance strategies), insecticides with a different mode of action should definitely be used, non-chemical protection measures should be applied and economic thresholds should be taken into account in control decisions [2,8,9,10]. This study, among others [2,11,12] explores the possibility of suppressing CPB through other measures, especially those that do not pose a threat to the environment, humans, and animals. Crop rotation, among the other cultural practices, is always recommended, and biological control measures can also be used.

Due to the resistance problem, there is a growing need for biological control for CPB. This pest has been shown to have a large number of natural enemies, for example the species *Perillus bioculatus* (Fab.) and *Podisus maculiventris* (Say) of the family Pentatomidae, which feed on CPB larvae. *Coleomegilla maculata* (DeGeer) attacks CPB eggs and larvae in the early stages of development. The species *Picromerus bidens* (L.) has also been successful in controlling CPB [13]. The species *Lebia grandis* (Hentz) of the family Carabide feeds on eggs, but also on the older larval stages, and develops an ectoparasitization on pupae. The parasite *Edovum puttleri* Griss. has successfully parasitized CPB eggs on eggplant [2]. The entomopathogen *Beauveria bassiana* (Bals.) Vuill. has an important role in the biological control of CPB. It can be applied directly to the soil or leaf surface [1].

Entomopathogenic nematodes (EPNs) can play an important role as biological control agents in integrated pest management strategies (IPM). Their natural habitat is soil and they live in moist conditions [14]. For biological control of CPB, the most promising EPN species belong to the Steinernematidae family. Today, the species *Steinernema carpocapse* (Weiser, 1955) and *Steinernema feltiae* (Filipjev, 1934) are commercialized for effective control of flies, weevils, mosquitoes, beetles, caterpillars, and other pests and are available as biological control agents [15].

The effectiveness of nematodes in controlling insect pests depends on biotic factors such as natural enemies and competition for resources. Among the natural enemies of EPNs, the most important are nematophagous fungi, Collembola, mites, and predatory nematodes may also be important [14]. Abiotic factors include their sensitivity to extreme temperatures, soil moisture, osmotic pressure, soil texture, pH and UV radiation [15].

Compared to conventional chemical insecticides, the use of EPNs has several advantages: fast and effective action, high reproductive potential, broad host list, they can be cultivated relatively easily, and they are not harmful to other living organisms or the environment [16]. In addition, there is no risk of residues, they are easy to apply, no waiting time is required from application to sowing or planting, they are compatible with other chemical products, and no protective equipment is required during application [9,17,18]. However, like any insecticide, EPNs have some disadvantages: broad host range (may include some beneficial insects), low tolerance to environmental conditions (e.g., moisture requirement), not suitable for long-term storage, non-persistent, and more expensive compared to chemical products [14,19,20].

The potential of using EPNs to control CPB and other pests has not yet been fully exploited. In Croatia, there are data on the potential use of *S. carpocapsae* for the control of codling moth (*Cydia pomonella* (L.)) [16] and the species *S. feltiae* for control of western corn rootworm (*Diabrotica virgifera virgifera* LeConte) [21], but there is not much information on their efficacy against CPB. Since they are ecotoxicologically safe, act quickly and efficiently over a long period of time, and are easy to apply [9], it would certainly be worthwhile to explore the possibility of suppressing Colorado potato beetle using EPNs, thus contributing to anti-resistance strategies to control this pest. Therefore, this study aimed to determine the efficacy of two nematode species (*Steinernema feltiae* and *Steinernema carpocapsae*) on adult Colorado potato beetles that had overwintered in the field where potatoes were grown the previous year.

## 2. Results

In spring 2018 and 2019, emergence of overwintered CPB adults was monitored in entomological cages for treatments with different doses of *S. feltiae* and *S. carpocapsae* and for the untreated control in fields where potatoes were grown in the previous year. In 2018, a total of 34 Colorado potato beetles were recorded, of which 20 individuals (58.82%) were found on the control, corresponding to an average infestation of 6.33 beetles/m^2^ or 63,300 beetles/ha, suggesting that pest intensity was high in the field studied.

The number of CPBs that emerged on the untreated control was similar in 2019 (23 individuals). However, the number of emerged beetles on treated plots was higher in 2019 than in 2018, suggesting that the efficacy of the applied treatments was lower in 2019 than in 2018.

### 2.1. Weather Conditions

**Figure 1:**
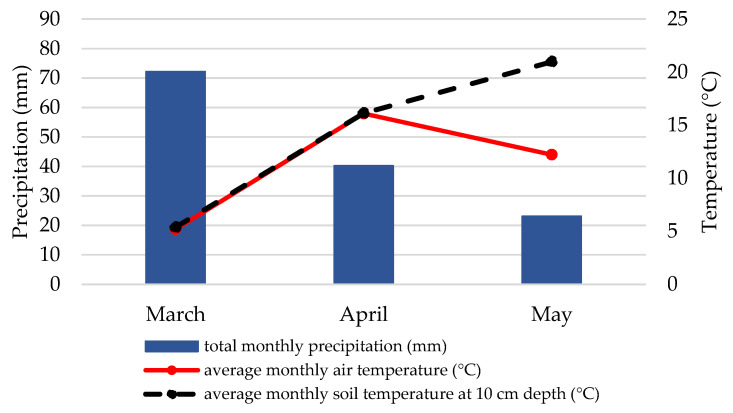
Weather conditions (the average monthly air temperature, total monthly precipitation and average monthly soil temperature at 10 cm depth) in March, April and May 2018, Zagreb. Data are presented up to 10 May 2018.

**Figure 2:**
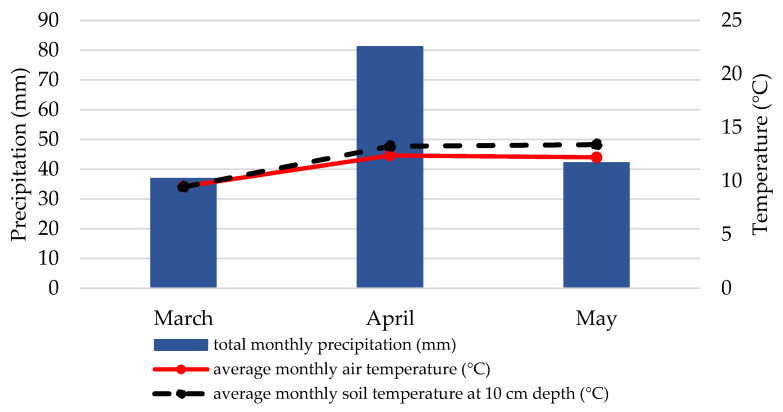
Weather conditions (the average monthly air temperature, total monthly precipitation and average monthly soil temperature at 10 cm depth) in March, April and May 2019, Zagreb. Data are presented up to 10 May 2019.

Weather data from the Croatian Meteorological and Hydrological Servicer recorded in March, April, and May 2018 and 2019 differed from year to year (Figure 1 and Figure 2). The average monthly air temperature in April and May was higher in 2018 than in 2019, which is also true for the soil temperature at 10 cm depth. Total monthly precipitation in April and May was almost half as much in 2018 as in 2019.

### 2.2. Efficacy of EPN Treatments

**Figure 3:**
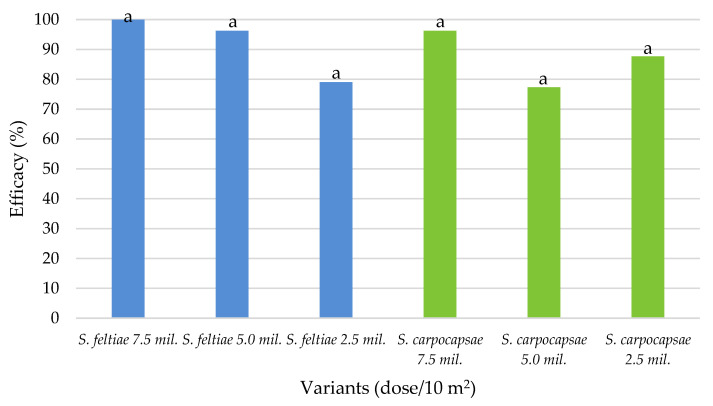
Average EPN efficacy on overwintered Colorado potato beetle populations in the 2018 field trial, Zagreb. Bars with the same letters are not significantly different (*p* < 0.05, Duncan’s New MRT test).

In 2018, both *S. feltiae* and *S. carpocapsae* showed good average efficacy on the overwintered CPB population, and no significant differences were observed between the different nematodes and the different dosages (Figure 3). Both EPNs had the highest efficacy at the highest dose applied, e.g., 100.00% for *S. feltiae* and 96.22% for *S. carpocapsae*. The recommended dose (5.0 mil./10 m^2^) for *S. feltiae* showed the same efficacy as the highest dose of *S. carpocapsae*, while the lowest dose of *S. feltiae* was slightly more effective (79.03%) as the recommended dose of *S. carpocapsae* (77.32%). The lowest dose of *S. carpocapsae* showed greater efficacy than the recommended dose, but the differences were not statistically significant.

**Figure 4:**
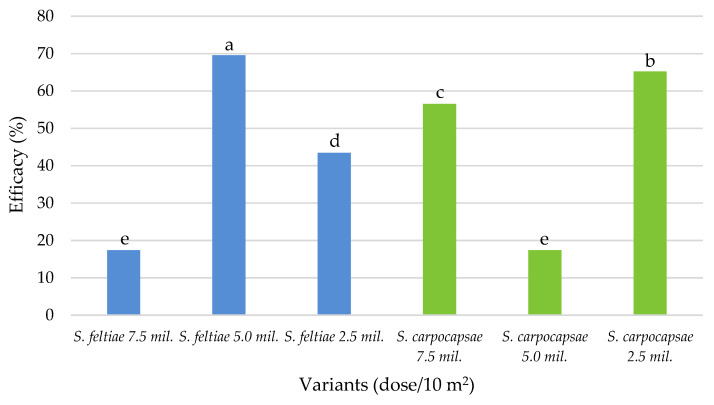
Average EPN efficacy on overwintered Colorado potato beetle populations in the 2019 field trial, Zagreb. Bars with different letters are significantly different (*p* < 0.05, Duncan’s New MRT test).

Statistical analysis of the results in the 2019 field trial showed significant differences in mean EPN efficacy between treatments (Figure 4). The highest efficacy (69.57%) was obtained with the recommended dose of *S. feltiae*, followed by the lowest dose of *S. carpocapsae* (65.22%). All other treatments showed significantly lower effect on reducing the number of overwintered adult CPB, as their efficacy ranged from 17.39% to 56.52%.

## 3. Discussion

In the literature review, several studies have shown that the nematode *S. feltiae* effectively suppresses CPB, but these are mainly studies of efficacy on larval stages rather than adults [22,23,24]. A study by Wright et al. [25] found high efficacy of different strains and doses of *S. feltiae* on larvae (80% to 90%) and overwintering CPB adults (88% to 100%). Kepenekci et al. [24] used the EPNs *S. feltiae, S. carpocapsae* and *H. bacteriophora* Poinar to control CPB larvae. *S. feltiae* caused 96% mortality, *H. bacteriophora* 75%, while *S. carpocapsae* caused only 36% mortality at the highest dose at 25 °C. Prishchepa et al. [26] studied the efficacy of S. *feltiae and S. carpocapsae* species on CPB and the results showed high efficacy of *S. feltiae* species while *S. carpocapsae* gave poorer results. A study conducted by Laznik et al. [27] showed that *S. feltiae* can be as effective as the chemical insecticide thiamethoxam on CPB larvae under field conditions. A similar study to ours, using entomological cages, was conducted by Toba et al. [28], in which no effect of *S. feltiae* on CPB adults was found, but later infection and mortality of larvae (59% to 71%, depending on the dose) were determined. Our study showed that there were no significant differences in efficacy between *S. feltiae* and *S. carpocapsae*. However, *S. feltiae* provided the highest efficacy value at the recommended dosages. The data also showed a large variability in efficacy from year to year.

Trdan et al. [29] conducted a study with four EPNs (*S. carpocapsae, S. feltiae, H. bacteriophora*, and *H. megidis* Poinar, Jackson & Klein) in CPB control. Mortality rate was influenced by temperature, nematode species, dose, and developmental stage of the pest. The highest mortality of adult individuals at temperatures between 20 °C and 25 °C was caused by *S. feltiae* and *S. carpocapsae*. At lower temperatures (15 °C), the highest dose of *S. feltiae* applied to overwintering adults showed high efficacy. *S. carpocapsae* was more effective at higher temperatures and is therefore recommended for control of first generation adult CPB. Similar results were obtained in the same study with larvae, as EPNs penetrate larval bodies more easily [29].

In addition to CPB, EPNs are also effective in controlling many other pests. For example, Canhilal and Carner [30] successfully suppressed the pest *Melittia cucurbitae* (Harris) with *S. feltiae, S. carpocapsae*, and *S. riobrave* Cabanillas, Poinar & Raulston, where *S. carpocapsae* and *S. feltiae* showed long infectivity in the soil after application. Batalla-Carrera et al. [31] showed that early larval stages of tomato leaf miner (*Tuta absoluta* (Meyrick)) were highly sensitive to the application of *S. carpocapsae, S. feltiae*, and *H. bacteriophora*. Even at a lower dose, *S. feltiae* showed the highest efficacy (100%), but other nematodes were also effective (*S. carpocapsae* 85.7%, *H. bacteriophora* 78.6%).

In our study, there was a difference in treatment efficacy between years that may have been influenced by weather conditions in the spring of 2018 and 2019 (Figure 1 and Figure 2). Although there are not many studies on the effects of weather conditions on CPB, Tauber et al. [32] and Lefevere and De Kort [33] found that precipitation has an impact on the occurrence of CPB. Their analyses showed that when precipitation decreases, CPB emergence from the soil slows down or stops altogether and can resume after sufficient precipitation and restoration of high soil moisture. The experimental field area received about half as much precipitation in April and May 2018 as in April and May 2019, and these conditions may have contributed to the low number of CPB beetles found in 2018. Another important factor that may have influenced the effectiveness of the treatments is soil temperature. In April and May 2018, the soil temperature ranged from 16 °C to 21 °C, while lower soil temperatures (13 °C in both April and May) were recorded in 2019. Radová and Trnková [34] studied the effect of soil temperature and moisture on the pathogenicity of the same two EPN species and reported that both *S. feltiae* and *S. carpocapsae* were significantly more efficient at temperatures between 15 °C and 25 °C, while at 10 °C their efficacy was very low. A similar effect was observed in our study, where efficacy was much higher in 2018 when soil temperature conditions were more favorable for the nematodes. In relation to precipitation (i.e., soil moisture content), *S. carpocapsae* is considered a species with better adaptation to dry conditions [35]. However, in studies [34], *S. feltiae* was found to tolerate dry conditions equally as well (in some cases even better) as *S. carpocapsae*. This may also help explain why both EPNs were not affected by the lower precipitation in 2018 and performed with high efficacy against CPB.

Considering that numerous studies on the control of CPB by these nematodes have been conducted primarily on larvae, our research provides a valuable contribution by demonstrating the possibility of suppressing the first occurrence of these important pests in the season. Often, overwintering adult CPB are not suppressed because they do not usually cause major damage to potatoes. However, suppression is justified when potatoes are weak and there are more than two beetles on a plant [8]. If this is the case, CPB should be controlled in fields where potatoes were grown the previous year and where CPB adults have overwintered, in order to reduce the abundance of the first generation and prevent the spread of new generations to surrounding potato growing areas. Such efforts could pose an organizational problem, since the same field may change ownership each year and the new owner may not need to use pesticides against overwintering Colorado potato beetle. Potato fields in Croatia are often smaller and not very far from each other, so overwintering CPB generations can easily spread to neighboring potato fields regardless of crop rotation. For control of overwintering CPB to be effective, an area-wide (AW) approach should be adopted, including larger potato growing areas. Drmić et al. [36] found area-wide mass trapping very helpful in reducing insecticide use and in establishing an integrated pest management plan to deal with sugar beet weevil. AW mass trapping in combination with other control measures also offers great potential for reducing codling moth damage levels [37]. In addition, insecticides with different modes of action should be used to reduce the potential for resistance to develop. More importantly, biological measures and biotechnical insecticides should be increasingly included. The use of locally available nematode strains should also be explored, as these are better adapted to local climatic conditions than commercial products and could provide better results.

Although a major disadvantage of entomopathogenic nematodes is their higher cost compared to chemical products [38], there are many other advantages: they act quickly and efficiently over a long period of time, have a broad host range, and can be easily applied. According to Oštrec [9], EPNs are not harmful to other living organisms and the environment, there is no risk of residues, there is no need to wait from application to sowing or planting, and no protective equipment is required during application. Based on the above facts, we can say that entomopathogenic nematodes are an environmentally friendly alternative to classical chemical control of CPB. As this research was carried out only on overwintering adults, it would be interesting and very valuable to investigate the EPN effect on other developmental stages of CPB in the course of potato cultivation, or, for example, to combine nematodes with other chemical or biological products.

## 4. Materials and Methods

### 4.1. Meteorological Data

Data collection on average daily air temperature, total daily precipitation and average daily soil temperature at 10 cm depth was obtained from the Croatian Meteorological and Hydrological Servicer in the period between March 1 and May 10 in both years for the weather station Maksimir, Zagreb.

### 4.2. Field Trial Setting and Treatments

This two-year (2018–2019) study was conducted at the Experimental Station Maksimir at the Faculty of Agriculture in Zagreb. The size of the field used in 2018 was 1800 m^2^, while the trial in 2019 was established on a field of 800 m^2^. The fields were located about 500 m apart. Potatoes were grown in both fields in the previous year, which had a natural infestation with Colorado potato beetle.

Two commercial products of entomopathogenic nematodes (EPNs) were used in the experiment, both manufactured by e-nema GmbH (Schwentinental, Germany) and supplied by Pro-eco (Varaždin, Croatia): Nemaplus^®^ containing the nematode *Steinernema feltiae*, and Nemastar^®^ containing *Steinernema carpocapsae*. The products were stored in a refrigerator (4–8 °C) for a few days until they were applied in the field.

The study included a total of seven treatments. The two commercial products were administered at three different doses: 7.5 million/10 m^2^, 5.0 million/10 m^2^ (the recommended dose), and 2.5 million/10 m^2^. Treatment 7 was the control in which only pure water was used. Each treatment was applied to an area of 2 m^2^ in three repetitions in a randomized block design.

The nematodes were applied as a liquid suspension on 4 April 2018 and 2 April 2019 by watering the soil early in the morning while the soil was still moist. The amount of water used was 1.5 L/m^2^. After treatment, the soil was watered with an additional 1 L of water/m^2^ as recommended by the manufacturer. To collect and monitor the number of overwintering adult Colorado potato beetles, an entomological emergence cage (1 m × 1 m × 1 m) was placed on each treatment on 6 April 2018 and 4 April 2019.

### 4.3. Assessment of Treatments and Statistical Analysis

Between 10 April and 10 May 2018, and between 6 April and 10 May 2019, the number of CPB adults that emerged in the entomological cages was recorded for each treatment. Surveys were conducted every 3–4 days and all collected beetles were removed from the cage during each inspection. Based on the total number of CPB that emerged on each variant, the efficacy of EPN treatments was calculated using the formula of Abbott [39].

To determine the effectiveness of EPNs between treatments in the study, the results were statistically analyzed by analysis of variance (ANOVA) and ranked by Duncan’s multiple range test (DMRT), using the statistical program ARM 9^®^ (GDM Solutions, Inc., Brookings, SD, USA) [40]. To assess normality, equality of variances was tested with Levene’s test and reaches with equal variances (*p* > 0.05). Efficacy calculations were available only for those treatments and assessment time points for which a statistically valid difference was found between the untreated control and the treatments in the study. In the case of uneven data distribution, data were transformed by log(x + 1) transformation.

## 5. Conclusions

The results of our two-year study show the potential use of EPNs for the control of Colorado potato beetle in Croatia. The EPNs *Steinernema feltiae* and *Steinernema carpocapsae* showed good overall efficacy in the field, although results in the second year of the trial were not as consistent as in the first year. Therefore, the study should be repeated on a larger number of fields and the efficacy should also be evaluated in different climatic areas of potato cultivation. Nevertheless, due to many advantages over chemical insecticides and the increasing problem of CPB resistance, EPNs represent a valuable tool and ecotoxicologically advantageous alternative to classical chemical control and their use may reduce the potential for CPB resistance development in potato growing areas in Croatia.

## Data Availability

Not applicable.
